# IL-1β in atherosclerotic vascular calcification: From bench to bedside

**DOI:** 10.7150/ijbs.66537

**Published:** 2021-10-22

**Authors:** Jialing Shen, Ming Zhao, Chunxiang Zhang, Xiaolei Sun

**Affiliations:** 1 Department of General Surgery (Vascular Surgery), the Affiliated Hospital of Southwest Medical University, Luzhou 646000, China.; 2 Department of Interventional Medicine, the Affiliated Hospital of Southwest Medical University, Luzhou 646000, China.; 3 Laboratory of Nucleic Acids in Medicine for National high-level talents, Southwest Medical University, Luzhou 646000, China.; 4 School of Cardiovascular Medicine and Sciences, King's College London British Heart Foundation Centre of Research Excellence, Faculty of Life Science and Medicine, King's College London, London SE5 9NU, United Kingdom.; 5 Centre for Clinical Pharmacology, William Harvey Research Institute, Barts and The London School of Medicine and Dentistry, Queen Mary University of London, London EC1M 6BQ, United Kingdom.; 6 Key Laboratory of Medical Electrophysiology, Ministry of Education & Medical Electrophysiological Key Laboratory of Sichuan Province, Collaborative Innovation Center for Prevention and Treatment of Cardiovascular Disease of Sichuan Province, Institute of Cardiovascular Research, Southwest Medical University, Luzhou 646000, China.; 7 Cardiovascular and Metabolic Diseases Key Laboratory of Luzhou, Luzhou, 646000, China.; 8 Nuclear Medicine and Molecular Imaging Key Laboratory of Sichuan Province, Luzhou 646000, China.

**Keywords:** IL-1β, vascular calcification, cardiovascular events, signaling pathways

## Abstract

Atherosclerotic vascular calcification contributes to increased risk of death in patients with cardiovascular diseases. Assessing the type and severity of inflammation is crucial in the treatment of numerous cardiovascular conditions. IL-1β, a potent proinflammatory cytokine, plays diverse roles in the pathogenesis of atherosclerotic vascular calcification. Several large-scale, population cohort trials have shown that the incidence of cardiovascular events is clinically reduced by the administration of anti-IL-1β therapy. Anti-IL-1β therapy might reduce the incidence of cardiovascular events by affecting atherosclerotic vascular calcification, but the mechanism underlying this effect remains unclear. In this review, we summarize current knowledge on the role of IL-1β in atherosclerotic vascular calcification, and describe the latest results reported in clinical trials evaluating anti-IL-1β therapies for the treatment of cardiovascular diseases. This review will aid in improving current understanding of the pathophysiological roles of IL-1β and mechanisms underlying its activity.

## Introduction

Atherosclerosis is initiated by disturbances in blood flow and numerous systemic factors including history of smoking, hypertension, diabetes, or hyperlipidemia 1-3. Atherosclerosis is part of the common pathophysiological basis for most cerebrovascular and cardiovascular diseases 4.

**Figure 1:**
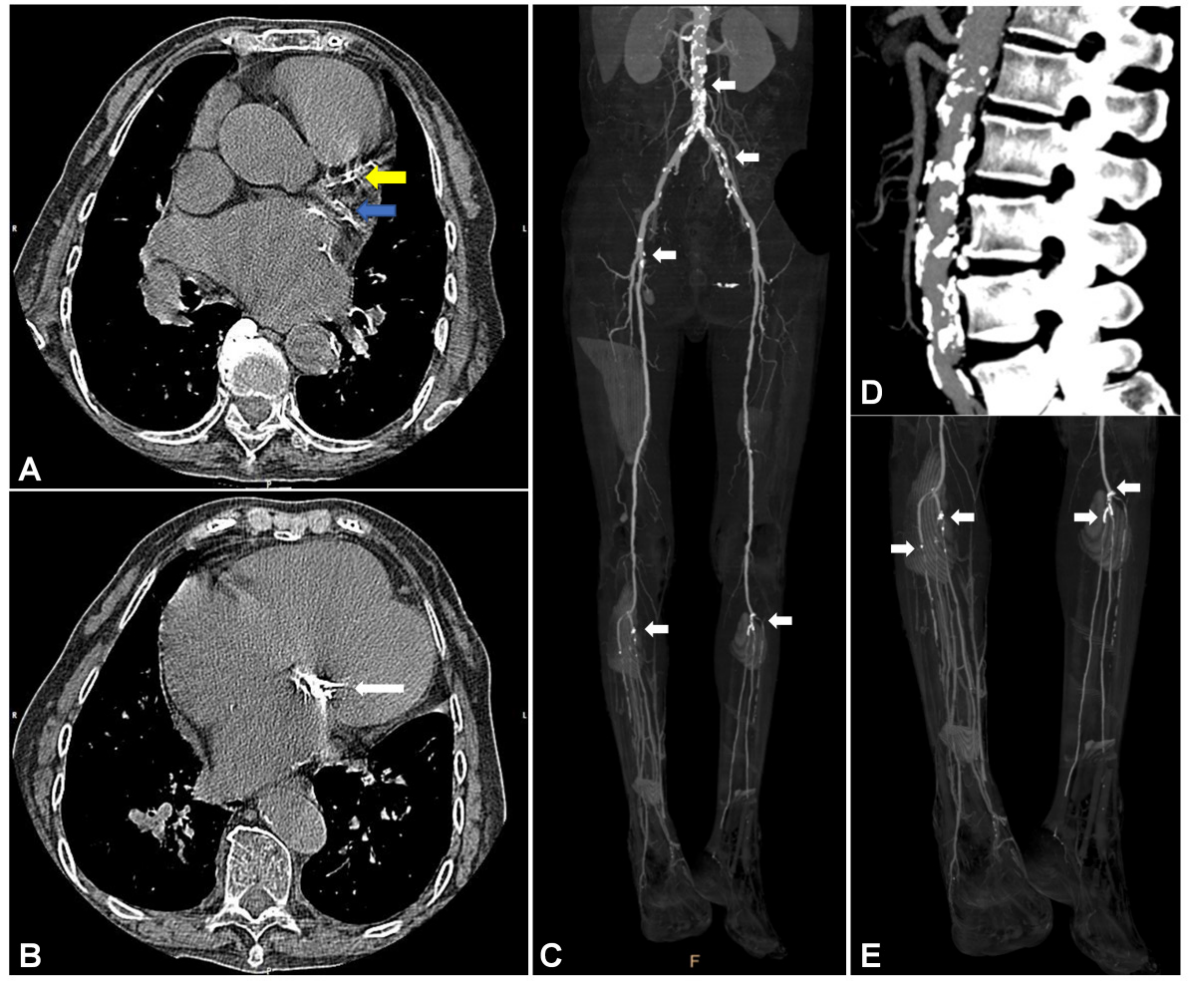
** Vascular calcification in cardiovascular vessels and valves.** (A) and (B) show vascular calcification in a 74-year-old woman with a history of rheumatic heart disease and hyperuricemia. (A) Representative CT image of vascular calcification in the left anterior descending (LAD, yellow arrow) and left circumflex artery (LCX, blue arrow) of the left coronary artery (LCA). (B) Representative CT image of vascular calcification in the mitral valve. Images (C), (D), and (E) show vascular calcification in a 56-year-old man with a history of arteriosclerosis obliterans (ASO), severe chronic limb ischemia accompanied by intractable and infectious ulcers, and type 2 diabetes mellitus (T2DM). (C) Representative infrarenal CT angiography (CTA) image shows multiple vascular calcifications. (D) Severe vascular calcification in the infrarenal aorta. (E) Severe vascular calcification in the distal popliteal artery and branches with stenosis and occlusion.

Vascular calcification is a ubiquitous pathological process in atherosclerosis 5, 6. The annual incidence of atherosclerotic vascular calcification in the general population ranges from less than 5% in individuals under 50 years of age to greater than 12% in individuals over 80 years of age 7. Imbalance in the calcium dynamics of atherosclerotic vessels can lead to reduced arterial compliance and impaired vascular hemodynamic responses 8, 9. Clinically, atherosclerotic vascular calcification (Figure 1) is implicated in aortic valve stenosis, congestive heart failure, myocardial infarction, peripheral arterial occlusion, and arterial hypertension 10, which lead to high rates of morbidity and mortality 11, 12.

Calcification of the aortic arch was first reported in relation to the risk of coronary heart disease in a cohort study examining 116,309 individuals with a median follow-up of 28-years 12. That study was also used to assess calcification of the coronary artery in asymptomatic individuals who experienced coronary events such as myocardial infarction or coronary death 11, and calcification of the abdominal aorta in individuals with increased risk for coronary heart disease 13. Under conditions of atherosclerosis, vascular calcification progresses, and is associated with cardiovascular events and increased mortality 7, 13-15. A meta-analysis examining 218,080 individuals with a mean follow-up of 10.1-years indicated that vascular calcification results in 4.63-fold higher risk for all-cause mortality, a 3.94-fold higher risk for cardiovascular mortality, and a 3.74-fold higher risk for any coronary events 15.

Although vascular calcification was previously considered passive and degenerative, it is currently recognized as an active and regulated pathobiological process. Vascular calcification, which shares many features with inflammatory atherosclerosis, may be treatable and preventable. The initial feature of calcified atherosclerotic vessels is activation of inflammation 16, 17. Cytokines secreted by inflammatory cells result in smooth muscle cell (SMC) apoptosis or SMC trans-differentiation into an osteochondrogenic cellular phenotype. Both of these events may contribute to mineral deposition in the plaque 18, 19. Among the numerous currently known inflammatory signaling pathways, those involving IL-1β are particularly implicated in atherosclerotic vascular calcification. In this review, we summarize the specific roles of IL-1β in atherosclerotic vascular calcification. This information will help delineate the pathogenesis of atherosclerosis, and will help uncover other, currently unknown, mechanisms involving IL-1β, in order to develop anti-IL-1β therapeutics for reducing the incidence of cardiovascular events 20.

## 1. Vascular calcification in atherosclerosis

Vascular calcification was previously believed to result from passive deposition of calcium and phosphorus on blood-vessel wall. Recent studies have shown, however, that vascular calcification is an active, reversible, highly regulated, and preventable process that is similar to physiological bone development 21. The two pathobiological mechanisms currently known to underlie vascular calcification are induction of osteogenesis and loss of inhibition of mineralization 22. Abnormalities in Ca^2+^ and phosphate metabolism 23, combined with increased oxidative stress 24, stimulation of inflammatory factors 25, dysregulation of certain miRNAs such as miR-34a 26, and disorders of lipid metabolism 27 lead to decreased expression of α-SMA, SM22α, and smooth muscle-myosin heavy chain, which are necessary for the maintenance of vascular function. Meanwhile, the expression of Runx2, SOX9, SP7, MSx2, and OPN, which are factors related to bone formation, is upregulated. This cascade promotes the activity of ALP and expression of BMP-2, resulting in osteogenic or chondral differentiation of VSMCs 8, 28. Inhibition of autophagy 29, matrix remodeling 30, cellular apoptosis, and matrix vesicles 31 may also accelerate vascular calcification. Recent studies in mouse models and humans have shown that blood vessels can produce and secrete factors, including PPI, OPN, OPG, MGP, FET-A, and Smad 6, which inhibit mineralization or vascular calcification 32-35. Inhibition of expression in these factors can lead to the initiation of vascular calcification.

Atherosclerosis usually leads to vascular calcification 36. In early stages of atherosclerosis, bone-related proteins can be detected histologically, and both the occurrence and development of vascular calcification are associated with the process of atherosclerosis 37. In the past decade, studies on coronary atherosclerotic calcification were mainly focused on lumen stenosis and plaque vulnerability. While calcification of atherosclerotic plaque core does not increase the vulnerability of the plaque 38, microcalcification of the fibrous caps on atherosclerotic plaques increases circumferential stress, which can, indeed, increase the vulnerability of the plaque. Overall, the size, shape, and location of the microcalcifications can directly define the vulnerability of the plaques 39. Electron beam computed tomography (CT) and intravascular ultrasound are currently used to detect calcifications in the arteries of 90% of patients with coronary atherosclerotic heart disease. Thus, the degree of vascular calcification may be directly related to the degree of vascular stenosis and risk for cardiovascular events in patients with atherosclerotic diseases 40.

Hence, an accurate, safe, and reproducible clinical detection technique is extremely important for the diagnosis, prevention, and treatment of vascular-calcification-related diseases. In the early stages of vascular calcification, molecular imaging technology, such as optical near-infrared fluorescence imaging 41, can be used to detect osteogenesis early at the (sub)cellular levels 42. The detection and quantification of advanced vascular calcification can be performed by CT, intravascular ultrasound (IVUS), transthoracic echocardiography, pulse wave velocity measurement, planar radiographs, and magnetic resonance imaging (MRI) 43. However, there are currently no satisfactory therapeutic approaches to vascular calcification in clinical practice. Even statins, which have been shown to decrease osteogenesis *in vivo* and *vitro* 44, 45, have failed to prove beneficial in clinical trials 46. Preventive measures are critical to decreasing the occurrence of vascular calcification and delaying the progression of vascular calcification. Although the modification of risk conditions (hyperglycemia, uremia, hypertension, hyperlipidemia, secondary hyperparathyroidism, and metabolic syndrome) and other factors (dietary phosphorous, oral activated charcoal, vitamins K and D, magnesium oxide, warfarin, bisphosphonates, antioxidants, estrogen, fetuin, osteopontin, anti-inflammatory agents, mineralocorticoids) might be possible 47-52, the details of any underlying mechanisms are not fully understood, and large-scale clinical trials have been limited.

## 2. Roles of the pro-inflammatory cytokine IL-1β

IL-1β is implicated in numerous inflammation-related diseases including rheumatoid arthritis, inflammatory bowel disease, osteoarthritis, type 2 diabetes, gout, multiple sclerosis, and Alzheimer's disease 53-55. As a potent, pro-inflammatory cytokine with a wide range of biological effects, IL-1β is synthesized and secreted by various cells including macrophages, fibroblasts, B lymphocytes, natural killer cells, and smooth muscle cells 56. As canonical negative feedback regulation to control IL-1β expression and secretion. IL-10 suppresses IL-1β and IL-1β-induced IL-1Ra 57, 58, the natural antagonist of IL-1β. There is also another mechanism underlying IL-1β-TGF-β1-related feedback to decrease the production of IL-1β 59, 60. One mechanism by which downstream and upstream negative feedback modulate IL-1β via paracrine secretion of interferons has been reported 61. Of which, IFN-II/IFNγ mediated the downstream regulation via its inhibition of the nitrosylation of NLRP3 inflammasome 62. While IFN-I/IFNβ mediated the upstream regulation via an IL-10 and STAT-3 dependent manner. The IFN-I/IFNβ secreted predominantly by fibroblasts responded to IL-1β elevation is a strong switch to attenuate the IL-1β induced inflammation at later stages 63. Recently, the secretion of IL-1β was revealed to be modulated by a negative feedback loop including IL-1β/NF-κB/TIR8/IL-1β during IL‐1β‐induced epithelial‐myofibroblast trans-differentiation 64.

IL-1β also plays an important role in the development of cardiovascular diseases 65. Various factors can activate IL-1β production and pyrolysis, enabling the participation of IL-1β in the pathophysiological process of cardiovascular diseases 66. Avolio *et al.* 67 and Qi *et al.* 68 discovered excessive activation of IL-1β in the hypothalamic paraventricular nucleus under conditions of hypertension, and found that inhibition of IL-1β can alleviate hypertension by reducing the activity of the sympathetic nervous system. Under conditions of hypertension, IL-1β also participates in the remodeling of aortic blood vessels by activating the renin-angiotensin-aldosterone system 69. Coronary artery thrombosis and blockade of coronary blood flow resulting from ruptured coronary artery plaques are the main causes of acute myocardial infarction. The ischemic or necrotic myocardium can generate increased levels of ATP and oxidative stress products, which then stimulate the expression of IL-1β 70, 71.

Atrial fibrillation is a common type of arrhythmia possessing a complex mechanism 72. Increased levels of IL-1β result in atrial myoelectrical and structural remodeling, and induction of atrial fibrillation 73. Activated Macrophage‐inducible C‐type lectin (Mincle) causes increased expression of IL-1β in the microglial cells residing in the paraventricular nucleus. At 24 hours after myocardial infarction, increased levels of IL-1β are increased further, and are accompanied by excessive activation of the sympathetic nerves. This cascade can result in the occurrence of a malignant ventricular arrhythmia 74.

Recent studies have shown that therapeutic targeting of inflammatory factors can improve cardiovascular outcomes in patients with a history of myocardial infarction 75. In the Canakinumab Anti-Inflammatory Thrombosis Outcomes Study (CANTOS), administration of the monoclonal IL-1β-neutralizing antibody canakinumab successfully reduced the rate of recurrent cardiovascular events by 17% 20. However, not all anti-inflammatory therapies benefit in the protection from cardiovascular events 75, 76, the detailed mechanism of anti-IL-1β-mediated activity remains unclear.

## 3. Signaling pathways involved in IL-1β-mediated regulation of atherosclerotic vascular calcification

### 3.1. IL-1β induces endothelial-to-mesenchymal transition and promotes atherosclerotic vascular calcification

Endothelial-to-mesenchymal transition (EndMT), a specific form of epithelial-to-mesenchymal transition (EMT), is characterized by the loss of endothelial features and acquisition of specific mesenchymal markers in endothelial cells 77, 78. EndMT is known to participate in the pathogenesis of atherosclerosis 79-82 and also occurs in atherosclerotic vascular calcification 83, 84.

IL-1β-mediated EndMT contributes to the pathogenesis of various diseases. Lee *et al.* have shown that injury-induced IL-1β expression induces EndMT in corneal fibrosis by upregulating the expression of FGF-2 85. Recombinant IL-1β induces EndMT in human esophageal microvascular endothelial cells, highlighting the important role of IL-1β in early-stage esophageal adenocarcinoma 86. IL-1β-induced EndMT also impairs the angiogenic potential of human umbilical vein endothelial cells (HUVECs) 87.

**Figure 2:**
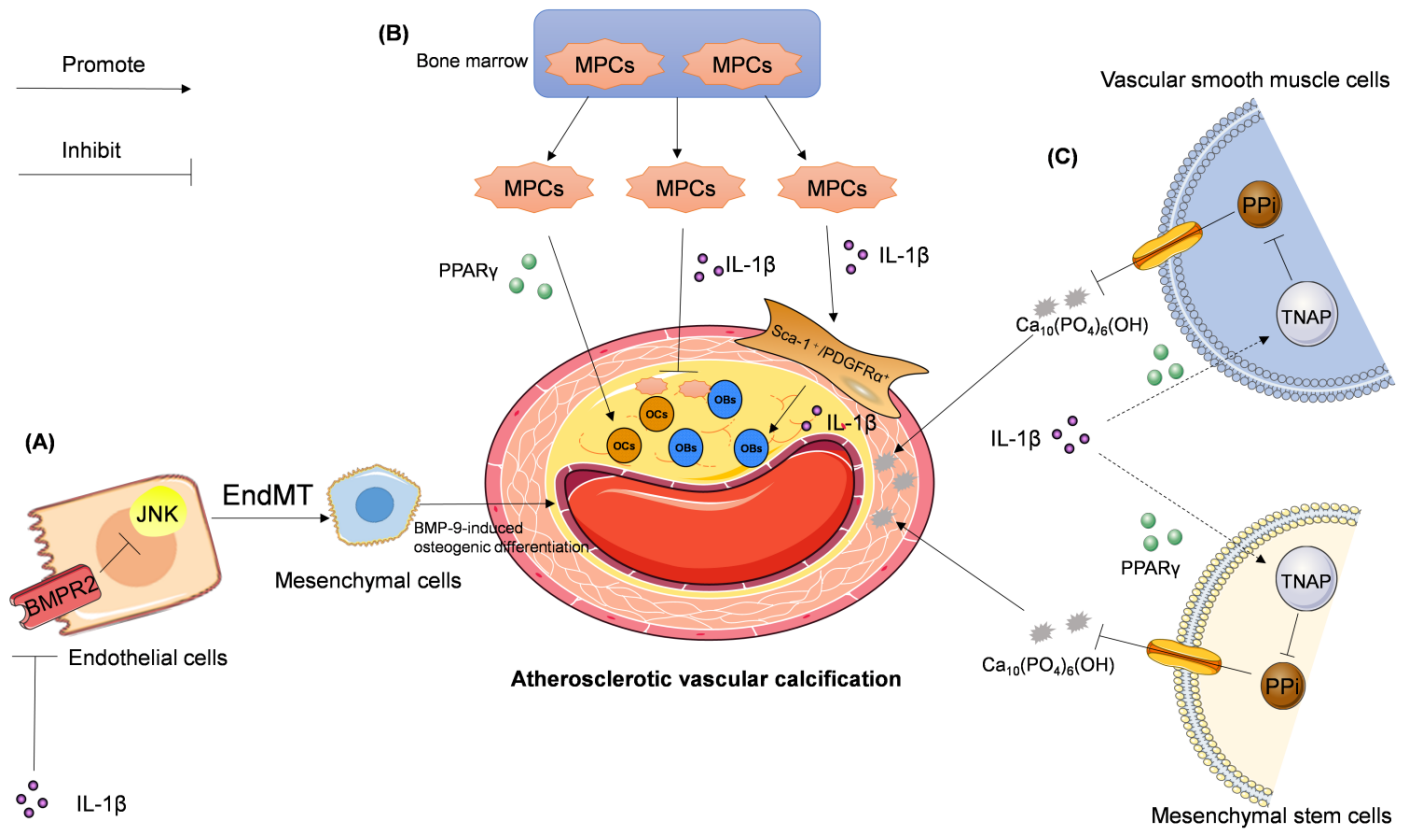
** Signaling pathways involved in IL-1β—mediated regulation of atherosclerotic vascular calcification.** (A) IL-1β activates JNK pathway by downregulating BMPR2 expression and subsequent BMPR2-dependent inhibition of JNK, which promotes endothelial to mesenchymal transition (EndMT), leading to BMP-9-induced osteogenic differentiation. (B) IL-1β inhibits the mobilization and infiltration of mesodermal progenitor cells (MPCs), which can bi-directionally differentiate into osteoblasts (OBs) or osteoclasts (OCs). While in hypercholesterolemia IL-1β enhances the mobilization and infiltration of Sca-1+/PDGFRα+ cells, which are differentiated from MPCs and the progenitor cells of OBs. PPARγ promotes the differentiation of MPCs into OCs. (C) IL-1β stimulates tissue-nonspecific alkaline phosphatase (TNAP) expression and activity in both vascular smooth muscle cells (VSMCs) and mesenchymal stem cells followed by pyrophosphate (PPi) degradation. PPi is an effective endogenous inhibitor of Ca_10_(PO_4_)_6_(OH), which is a major component of the calcified aorta. This chain of events accelerates atherosclerotic vascular calcification.

The relationship between IL-1β and EndMT in atherosclerotic vascular calcification is still poorly understood. Sanchez-Duffhues *et al.* reported that IL-1β-sensitized bone morphogenetic protein-9 (BMP-9)-induces osteogenic differentiation via induction of EndMT. This process is mediated by the downregulation of bone morphogenetic protein receptor type II (BMPR2) expression and subsequent inactivation of the c-Jun N-terminal kinase (JNK) signaling pathway in human primary aortic endothelial cells 88. This hypothesis was further corroborated *in vivo* and in patient-derived atherosclerotic tissues 88. These findings suggest that IL-1β may induce EndMT and promote atherosclerotic vascular calcification (Figure 2A).

### 3.2. IL-1β inhibits the mobilization and infiltration of bipotent mesodermal progenitor cells (MPCs), thereby accelerating atherosclerotic vascular calcification

Stem or progenitor cells, and their dynamics, play an important role in cardiovascular diseases 89-92. Using identification of cell surface markers, such as platelet-derived growth factor receptor alpha (PDGFRα) and stem cell antigen-1 (Sca-1), Cho *et al.* 93 discovered a cluster of vascular calcifying progenitor cells residing in the arterial adventitia. These cells are derived from the bone marrow and mobilize to the inflamed atherosclerotic lesions. Mesodermal progenitor cells (MPCs), such as Lin-CD29+/Sca-1+/PDGFRα- cells, possess bidirectional differentiation, which enables the development of MPCs into osteoblasts (OBs) or osteoclasts (OCs). MPCs isolated from the adult bone marrow are also progenitors of Sca-1+/PDGFRα+ cells, which can potentially differentiate into OBs 94. IL-1β, which is elevated in the sera and arteries of hypercholesterolemic *ApoE^-/-^* mice, enhances the mobilization and infiltration of Sca-1+/PDGFRα+ cells. Conversely, IL-1β inhibits bipotent MPCs and accelerates atherosclerotic vascular calcification 94. Thus, current studies suggest that IL-1β is likely a key regulator of bipotent MPCs in vascular calcification.

Activation of peroxisome proliferator-activated receptor γ (PPARγ) promotes the differentiation of bipotent MPCs into OCs; this process alleviates vascular calcification *in vitro* and *in vivo* 93. Collectively, the homeostasis between MPCs and Sca-1+/PDGFRα+ cells may play an important role in vascular calcification under conditions of atherosclerosis (Figure 2B). Induced pluripotent stem (iPS) cells originating from MPC-related cells 95, and monoclonal antibodies specific for IL-1β 96-98, are currently available as sources for the development of new therapeutics for the treatment of patients with atherosclerotic vascular calcification 93.

### 3.3. IL-1β activates tissue-nonspecific alkaline phosphatase to exacerbate atherosclerotic vascular calcification

Tissue-nonspecific alkaline phosphatase (TNAP) is an enzyme that degrades extracellular pyrophosphate (PPi) and promotes vascular calcification by downregulating the expression of PPi. PPi is a potent endogenous inhibitor of hydroxyapatite [Ca_10_(PO_4_)_6_(OH)] 99-101, which is the main component of the calcified aorta 102. This decreased level of plasma PPi is associated with genetic or metabolic conditions, such as Hutchinson-Gilford progeria syndrome (HGPS) 99, generalized arterial calcification of infancy (GACI) 103, and advanced chronic kidney disease 104, which predispose vulnerable individuals to vascular calcification 105-107. Transgenic overexpression of TNAP in VSMCs or in endothelial cells results in pathological calcification *in vitro* and *in vivo* 108-110. Moreover, the upregulation of TNAP expression has also been demonstrated in dialysis-related calcification of human vessels 111.

Ding *et al.* 112 reported that IL-1β can upregulate TNAP activity and calcification in human mesenchymal stem cells via RUNX2-independent signaling. Lencel *et al.* 113 utilized VSMCs, stimulated by TNF-α and IL-1β, to demonstrate the cell-specific effects of TNAP, and suggested that PPARγ may mediate these differences in TNAP activity. For these reasons, IL-1β is considered a stimulator of vascular calcification in the context of atherosclerosis. Few studies have examined the roles of IL-1β and TNAP in vascular calcification. However, whether IL-1β can promote vascular calcification via a TNAP-mediated signaling pathway needs further study (Figure 2C).

## 4. Stimulators that regulate IL-1β expression accelerate atherosclerotic vascular calcification

### 4.1. Rac2 mediates atherosclerotic vascular calcification via regulation of IL-1β production in macrophages

**Figure 3:**
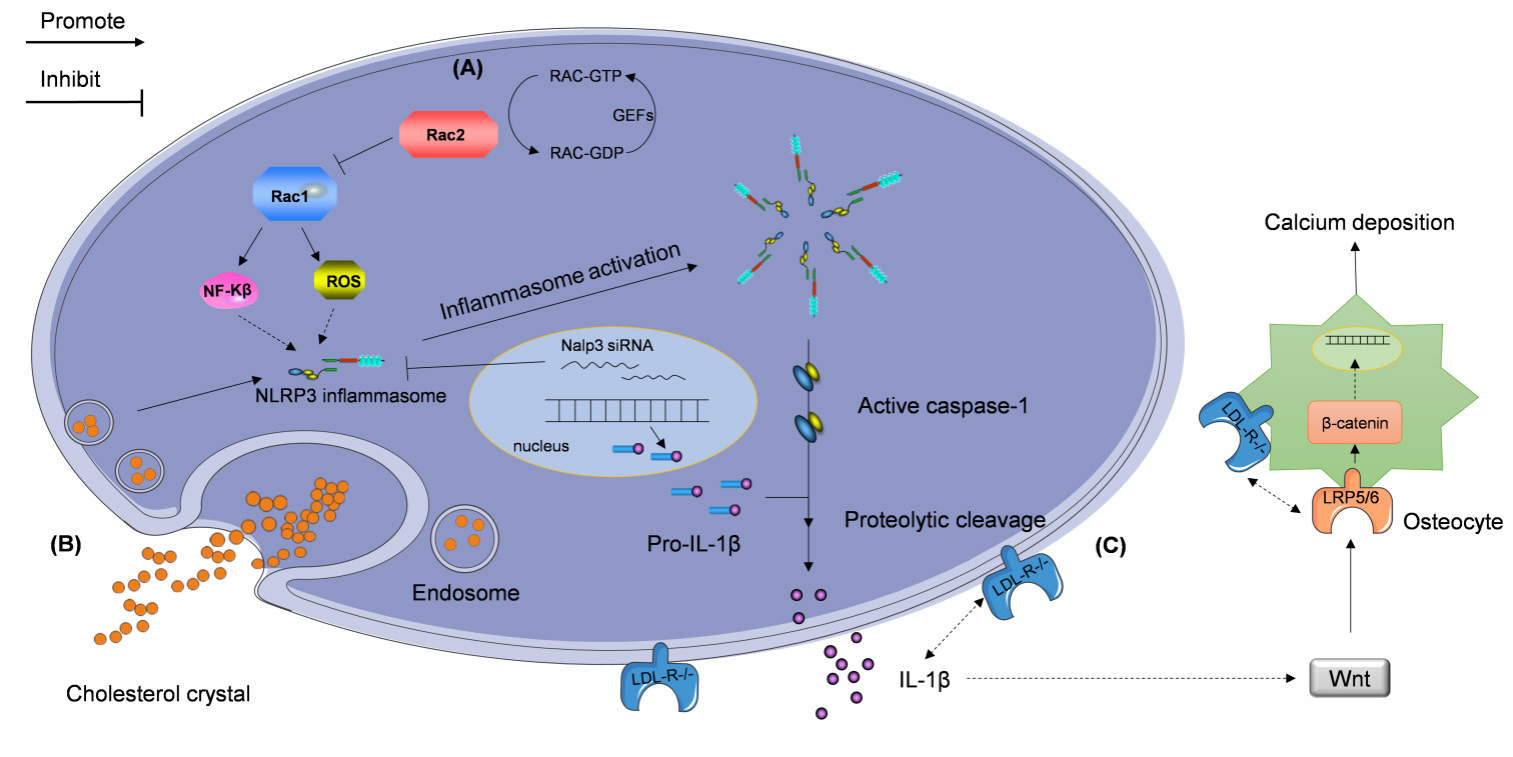
** Stimulators regulating IL-1β expression accelerate atherosclerotic vascular calcification.** The NLRP3 inflammasome participates in the regulation of the progression of atherosclerotic vascular calcification via two signaling pathways described as follows. (A) The reduced Rac2 activity, caused by compromised guanine nucleotide exchange factors (GEFs), elevates the expression of Rac1, which promotes activation of NF-κB pathway and production of reactive oxygen species (ROS). These events lead to the NLRP3 inflammasome production. (B) Extracellular cholesterol crystals are taken up by macrophages and activate the NLRP3 inflammasome. NLRP3 inflammasome-activated caspase-1 cleaves pro-IL-1β and induces the secretion of IL-1β. (C) Deletion of the gene encoding low-density lipoprotein receptor (LDL-R) contributes to elevation in IL-1β expression and subsequent vascular calcification independent of hypercholesterolemia. This cascade may be modulated by the Wnt and β-catenin signaling pathways.

Rac proteins are a subfamily of the Rho family, which consists of small guanosine triphosphate-binding proteins Rac 1, 2, 3, and RhoG 114, 115. Similar to other small GTPases, Rac switches between a GTP-bound active and GDP-bound inactive state. Stimulus-induced activation of Rac is mediated by guanine nucleotide exchange factors (GEFs) 116-118; this subfamily of proteins is critical in numerous inflammation-mediating pathological processes. Rac1 and Rac2, which are important signal transducers in inflammatory cells, affect the expression of several cytokines and growth factors 119, 120. Recently, Ceneri *et al.* 121 reported a significant decrease in Rac2, and increase in IL-1β, expression, in the aortas of *ApoE^-/-^* mice and in calcified plaques in human coronary segments. They also showed that Rac2 deletion can aggravate vascular calcification by increasing macrophage expression of IL-1β, which is associated with NF-κB activation. Rac2 was also found to enhance the production of reactive oxygen species (ROS) elicited by elevation of activated Rac1 expression in *Rac2^-/-^ApoE^-/-^* mice. Thus, Ceneri *et al.* identified a novel inflammatory signaling pathway that depends on Rac2-mediated regulation of Rac1-dependent IL-1β expression in macrophages (Figure 3A) 121. This study outlines a pathophysiological signaling mechanism to understand the rationale of the action of IL-1β in the development of atherosclerotic vascular calcification.

### 4.2. NLRP3 inflammasome initiates the release of IL-1β by upregulating the expression of caspase-1, leading to atherosclerotic vascular calcification

The NLR family pyrin domain-containing 3 (NLRP3) inflammasome, which is an ROS-sensitive multiprotein complex, accelerates IL-1β maturation via activation of caspase-1 122, 123. NLRP3 plays a pivotal role in the pathogenesis of atherosclerosis 124-126. Numerous studies have reported that NLRP3 is essential for atherogenesis, and that silencing the expression of NLRP3 prevents the rupture of atherosclerotic plaques 127, 128.

Wen *et al.* 129 found that the expression of NLRP3 inflammasome and IL-1β are upregulated in VSMCs cultured in calcification medium supplemented with β-glycerophosphate (β-GP) and in human calcified popliteal arteries having elevated expression of caspase-1. Downregulation of NLRP3 expression using treatment with NLRP3 siRNA reduces IL-1β secretion, which subsequently reduces vascular calcification, *in vitro*. However, whether the knockdown of IL-1β can counteract the effect of NLRP3 inflammasome in vascular calcification remains unclear and needs to be studied *in vivo*. Tangi *et al.* 130 demonstrated that TNF-α-mediated induction of IL-1β release in aortic smooth muscle cells is also NLRP3-dependent. Additionally, the NLRP3 inflammasome-mediated promotion of IL-1β secretion via upregulation of caspase-1 expression may play an important role in atherosclerotic vascular calcification (Figure 3B). Further investigation is required to uncover the details of this mechanism.

### 4.3. Hypercholesterolemia promotes the release of IL-1β to induce atherosclerotic vascular calcification

The effect of hypercholesterolemia on vascular calcification was confirmed when Awan *et al.* 131 reported that patients with familial hypercholesterolemia (FH), who possess mutations in the low-density lipoprotein receptor (*LDLR*) gene, show severe and extensive vascular calcification in their thoracoabdominal aortas; this process usually commences at 20 years of age in homozygous FH patients. In contrast, vascular calcification in thoracoabdominal aorta is delayed by two decades in individuals affected by heterozygous FH 132. Although a comparably high level of plasma cholesterol is observed in *Ldlr^-^/^-^*C57BL/6 mice fed a standard-chow diet and wild-type C57BL/6 mice fed a high-cholesterol, high-fat diet, *Ldlr^-^/^-^* mice still develop considerably more extensive aortic vascular calcification compared with that of wild-type mice 133. None of the currently available therapeutics, including statins, can stop or regress vascular calcification in a clinical setting 134. Collectively, these findings suggest that vascular calcification in early hypercholesterolemia may involve as of yet undiscovered mechanisms, and that LDLR deficiency may be a key step in vascular calcification.

Duewell *et al.* 125 and Rajamaki *et al.* 135 found that crystals of cholesterol are absorbed by macrophages, which then express the activated inflammasome complex. These events result in cleavage and activation of pro-IL-1β and secretion of IL-1β into plasma. Consequently, the binding of plasma IL-1β to IL-1β receptors on vascular endothelial cells promotes the release of various cytokines, SMC proliferation, and macrophage activation. The steps in this cascade contribute to the development of atherosclerosis 136, but the mechanism underlying hypercholesterolemia and IL-1β activity in the context of vascular calcification remains to be uncovered.

The levels of total cholesterol are increased in LDLR-deficient (*Ldlr^-^/^-^*) mice and LDLR-attenuated proprotein convertase subtilisin/kexin type 9 (Pcsk9) transgenic (Tg) mice 137. However, Awan *et al.* 138 reported that IL-1β plasma levels in *Ldlr^-^/^-^* mice are twice as high as those in Pcsk9(Tg) mice. They also found that while anti-IL-1β monoclonal antibodies considerably inhibit atherosclerotic vascular calcification in *Ldlr^-^/^-^* mice with hypercholesterolemia, they induce only an insignificant change in the atherosclerotic vascular calcification of Pcsk9(Tg) mice with indiscriminate hypercholesterolemia. This finding suggests a potential mechanism accounting for why Pcsk9(Tg) mice show significantly less calcification compared with that in *Ldlr^-^/^-^* mice. This finding indicates that IL-1β plays a key role in atherosclerotic calcification associated with LDLR deficiency. Although a hypothesis linking IL-1β, LDL-R, and the Wnt/β-catenin signaling pathways to vascular calcification in the setting of hypercholesterolemia has been proposed 133, the detailed mechanism is unknown and further investigation is still needed (Figure 3C).

## 5. Discussion

Inflammation is proved to be an independent risk factor for cardiovascular disease, even after the lipid-lowering therapies. The SPIRE-1 and SPIRE-2 trials 139 using the statins and PCSK9 antibody bococizumab to reduce atherogenic lipids showed residual inflammation and no improvement for cardiovascular events. Since 2018, several anti-inflammation clinical trials revealed promising and challenging results for the treatment of cardiovascular disease, such as CANTOS 20 and CIRT 76. The CANTOS trial has shown that treatment with the monoclonal IL-1β-neutralizing antibody canakinumab can reducing the risk of recurrent cardiovascular events in patients with prior heart attack. While the CIRT trial in 5,000 patients with previous coronary disease showed no benefit for the reduction of cardiovascular events with the treatment of methotrexate—an promising anti-inflammatory approach in which once an association with fewer cardiovascular events was observed in patients with rheumatoid. These results provide us with informative implications that more unknown mechanisms exist in the anti-inflammation therapeutics for the prevention of cardiovascular events. A hypothesis of 'innate immune training' of IL-1β in epigenetic reprogramming of myeloid progenitor cells 140 was once proposed to understand the mechanism of the action of IL-1β. However, disputes of this issue exist 75.

Accumulating clinical evidence indicates that vascular calcification is an independent predictor of the occurrence of atherosclerosis-related myocardial infarction and stroke 7, 11-13, 15, 141-143. Recent studies, including those summarized in this review, have shown that IL-1β plays prominent roles in the development of atherosclerotic vascular calcification. Although the incidence of atherosclerosis-related cardiovascular events has been clinically reduced by the use of anti-IL-1β therapy, as shown in several large-scale population cohort trials (Table 1), the clinical effect of IL-1β inhibition on atherosclerotic vascular calcification remains unclear. It is also unclear whether the beneficial effect induced by anti-IL-1β therapeutics in the setting of atherosclerosis is related to the effect of these therapeutics on atherosclerotic vascular calcification.

Further prospective randomized controlled trials (RCTs) are needed to evaluate the effects of IL-1β-related antibodies and drugs (Table 2) on atherosclerotic vascular calcification and incidence rate of cardiovascular events. Even in the well-known CANTOS trial 20, 144, there is no subgroups treated with anti-IL-1β therapy showing variance in the levels of vascular calcification in the setting of cardiovascular events. Therefore, further clinical trials, especially RCT studies, are needed to explore the roles of IL-1β and related therapeutics in atherosclerotic vascular calcification.

*IL1B^-/-^*, *IL1R1^-/-^*, IL-1 receptor antagonist-deficient (*IL1Ra*^-/-^), and transgenic mice overexpressing either secreted IL-1Ra or intracellular IL-1Ra1, are valuable animal models employed in basic *in-vivo* and *in-vitro* research 145-150. These models are useful to mimic cardiac infarction and ischemic stroke in order to uncover the mechanism linking IL-1β activity, atherosclerotic vascular calcification, and cardiovascular events. New technologic advances, such as using artificial intelligence to conduct big-data analysis, will help us delineate the detailed mechanisms of cardiovascular calcification and design potential therapeutics 151. These future studies will provide us with a broad understanding of IL-1β roles in atherosclerotic vascular calcification, and will define the value of IL-1β as a potential therapeutic target in the treatment of patients with cardiovascular diseases.

In summary, atherosclerotic vascular calcification is a vascular lesion related to morbidity and mortality worldwide. Currently, no specific and effective treatments are available for this condition because of our insufficient understanding of the detailed molecular mechanisms driving the processes of atherosclerotic vascular calcification. Future research should aim to delineate these molecular mechanisms, including those involving IL-1β-mediated pathways, as related to the development of atherosclerotic vascular calcification.

## Figures and Tables

**Table 1:** Clinical trials examining anti-IL-1β therapy for treatment of patients with cardiovascular diseases

Trials	Drug type	Dosage and administration	Treatment duration	Outcomes
Virginia Commonwealth University-Anakinra Remodeling Trial (VCU-ART) 152	Recombinant IL-1 receptor antagonist (IL-1Ra)	Anakinra, 100 mg/day, subcutaneous injection	14 days	Double-blinded, randomized, placebo-controlled; 10 patients with ST-segment elevation of acute myocardial infarction (AMI). Anakinra improved the end-diastolic volume index and left ventricular end-systolic volume index (LVESVi).
Virginia Commonwealth University-Anakinra Remodeling Trial 2 (VCU-ART2) 153	Recombinant IL-1 receptor antagonist (IL-1Ra)	Anakinra, 100 mg/day, subcutaneous injection	14 days	Double-blinded, randomized, placebo-controlled; 30 patients with stable ST-segment elevation of acute myocardial infarction (AMI). Anakinra had no effect on LVESVi and end-diastolic volume index, but significantly alleviated the upregulation of C-reactive protein and lowered the occurrence of heart failure. Data pooled from the above VCU-ART and VCU-ART2 trials revealed that anakinra reduced the incidence of primary heart failure in contrast with that in the placebo-treated group.
MRC-ILA Heart Study 154, 155	Recombinant IL-1 receptor antagonist (IL-1Ra)	Anakinra, 100 mg/day, subcutaneous injection	14 days	Phase II, double-blinded, randomized, placebo-controlled; 182 patients diagnosed with non-ST elevation acute coronary syndrome (NSTE-ACS), admitted to hospital <48 h from the development of chest pain. Anakinra reduced the high-sensitivity C-reactive protein levels at 7 days post-treatment.
Canakinumab Anti-Inflammatory Thrombosis Outcomes Study 20, 144	Monoclonal IL-1β-neutralizing antibody	Canakinumab, 50 mg, 150 mg, and 300 mg, subcutaneous injection, every 3 months, respectively	48 months	A randomized, double-blinded trial enrolling 10,061 patients. Canakinumab, administered at the dose of 150 mg, reduced the rate of recurrent cardiovascular events by 17%.

**Table 2:** IL-1β-related antibodies and drugs

Names	Type	Application
XOMA052 156, 157	Monoclonal anti-IL-1β antibody	*In vitro* and *in vivo*
Gevokizumab 158	IL-1β modulator	*In vitro* and *in vivo*
Anakinra 152-155	Recombinant IL-1 receptor antagonist (IL-1Ra)	Clinical trial
Canakinumab 20, 144	Monoclonal IL-1β-neutralizing antibody	Clinical trial

## Abbreviations

ALP: Alkaline phosphatase
AMI: Acute myocardial infarction
ASO: Arteriosclerosis obliterans
BMP-2: Bone morphogenetic protein-2
BMP-9: Bone morphogenetic protein-9
BMPR2: Bone morphogenetic protein receptor type II
CACS: Coronary artery calcium scoring
CANTOS: Canakinumab Anti-Inflammatory Thrombosis Outcomes Study
CTA: Computed tomography angiography
EndMT: Endothelial-to-mesenchymal transition
EMT: Epithelial-to-mesenchymal transition
Fet-A: Fetuin A
FH: Familial hypercholesterolemia
GACI: Generalized arterial calcification of infancy
GEFs: Guanine nucleotide exchange factors
HGPS: Hutchinson-Gilford progeria syndrome
HUVECs: Human umbilical vein endothelial cells
IL-1β: Interleukin-1β
IL-1Ra: IL-1 receptor antagonist
iPS: Induced pluripotent stem
JNK: c-Jun N-terminal kinase
LAD: Left anterior descending
LCA: Left coronary artery
LCX: Left circumflex artery
*LDLR*: Low-density lipoprotein receptor
*Ldlr^-^/^-^*: LDLR-deficient
LVESVi: Left ventricular end-systolic volume index
MGP: Matrix Gla protein
Mincle: Macrophage‐inducible C‐type lectin
MPCs: Mesodermal progenitor cells
MSx2: Muscle segment homeobox homolog of 2
NLRP3: Nlp family, pyrin domain‐containing 3
NSTE-ACS: Non-ST elevation acute coronary syndrome
OB: Osteoblastic
OC: Osteoclastic
OPN: Osteopontin
OPG: Osteoprotegerin
Pcsk9: Proprotein convertase subtilisin/kexin type 9
PDGFRα: Platelet-derived growth factor receptor alpha
PPARγ: Peroxisome proliferator-activated receptor γ
PPi: Pyrophosphate
RCTs: Randomized controlled trials
Runx 2: Runt-related transcription factor 2
Sca-1: Surface markers stem cell antigen-1
Smad 6: Drosophila mothers against decapentaplegic protein 6
SMC: Smooth muscle cell
Sox 9: SRY-Box transcription factor 9
SP 7: Osterix
T2DM: Type 2 diabetes mellitus
Tg: Transgenic
TNAP: Tissue-nonspecific alkaline phosphatase
TNF-α: Tumor necrosis factor-α
VSMCs: Vascular smooth muscle cells
